# Wolf Delisting Challenges Demonstrate Need for an Improved Framework for Conserving Intraspecific Variation under the Endangered Species Act.

**DOI:** 10.1093/biosci/biaa125

**Published:** 2020-10-29

**Authors:** Carlos Carroll, Daniel J Rohlf, Bridgett M vonHoldt, Adrian Treves, Sarah A Hendricks

**Affiliations:** Klamath Center for Conservation Research, Orleans, California; Earthrise Law Center, in the Lewis and Clark Law School, Portland, Oregon; Department of Ecology and Evolutionary Biology, Princeton University, Princeton, New Jersey; Nelson Institute for Environmental Studies, University of Wisconsin, Madison, Wisconsin; Institute for Bioinformatics and Evolutionary Studies, University of Idaho, Moscow, Idaho

**Keywords:** adaptive potential, Canis lupus, conservation genomics, distinct population segment, recovery planning

## Abstract

Recent advances in genomics have increased our understanding of geographic patterns of intraspecific variation and the importance of this variation in enhancing species’ potential to adapt to novel threats. However, as part of an effort to limit the scope of the Endangered Species Act (ESA), the US government has proposed the removal of the gray wolf from the list of protected species on the basis of a claim that the statute permits a species to be declared recovered given the existence of a single presently secure population. We rebut this interpretation and propose a framework for the conservation of adaptive potential that builds on current agency practice in delineating subspecific recovery units and reconciles the definition of significance in the statute's “distinct population segment” and “significant portion of range” clauses. Such a coordinated policy would enhance the ESA's effectiveness in stemming loss of biodiversity in the face of climate change and other factors altering Earth's ecosystems.

Although the US Endangered Species Act (ESA; 16 U.S.C. §§ 1531–44) is among the world's most influential biodiversity protection statutes, key aspects of how the law should be implemented remain contested. A central issue involves the appropriate level of ambition for recovery of formerly widely distributed species such as the North American gray wolf (*Canis lupus*; figure 1; Enzler and Bruskotter 2009, Carroll et al. 2010). If the ESA aims only to prevent the complete extinction of a species, is the existence of a single secure population sufficient to declare a species recovered? Alternately, does a species need to achieve recovery in all or a majority of its historical range before it can be removed (delisted) from the list of protected species? If the purpose of the statute lies somewhere between these bounds, how can appropriate recovery goals be established? These questions resonate beyond the US context because they address how best to conserve variation below the level of the taxonomic groupings (species and subspecies) typically acknowledged in conservation statutes of other nations (Laikre et al. 2016, vonHoldt et al. 2018, Hendricks et al. 2019a).

**Figure 1.**
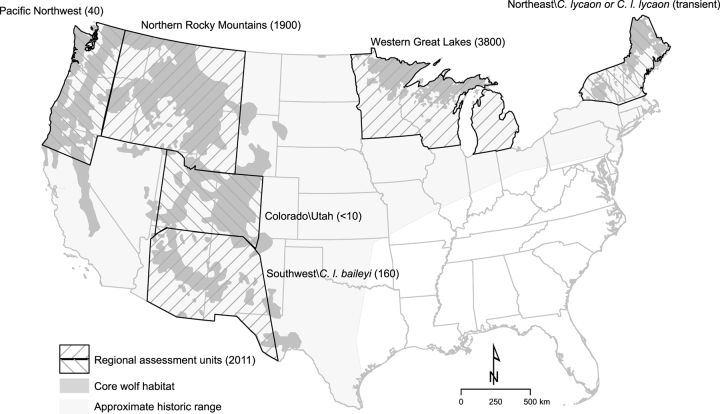
Map of regional assessment units used in the 2008–2011 national wolf strategy process (Runge 2011). Current estimates of population numbers in each assessment unit (USFWS 2019) are given in parentheses and are approximate particularly in two units (Western Great Lakes and Northern Rocky Mountains) with recent changes in census methods. Wolf packs in Washington and Oregon are divided between the Pacific Northwest and Northern Rocky Mountain assessment units, with most falling within the latter unit. Distribution of potential core habitat is as delineated by CBD and HSUS (2018) based on published regional habitat models. Many areas of potential core habitat currently lack wolves, and many areas of historical range outside of core habitat could be inhabited by wolves given sufficiently low anthropogenic mortality.

Although Congress and federal agencies have long recognized the importance of conserving intraspecific variation, recent agency actions, exemplified by a 2019 proposal to delist the gray wolf (84 FR 9648), suggest a shift away from biologically informed policy (Lambert 2019). In this Forum, we use the 2019 delisting proposal to demonstrate that recent inconsistent implementation of the ESA's mandate for the conservation of intraspecific variation undermines the conservation outcomes intended by Congress. We propose a more consistent and transparent framework that coordinates the two elements of the ESA that authorize the conservation of intraspecific variation: the distinct population segment (DPS; see supplemental table S1 for a definition of terms) and significant portion of range (SPR) clauses, while building on current agency guidance for delineating subspecific recovery units. Rather than representing a detailed policy proposal or a comprehensive review of case law in the present article, we synthesize information from the fields of conservation genetics, wildlife ecology, and endangered species law to advance the discussion and resolution of conceptual issues regarding the conservation of intraspecific variation under the ESA.

## Why is conservation of intraspecific variation important?

Why would a statute designed to protect the nation's biodiversity, such as the ESA, mandate the conservation of multiple populations of widely distributed species rather than a museum piece approach (Vucetich and Nelson 2014) based on preserving a single narrowly distributed population? The ESA's preamble mentions an array of “esthetic, ecological, educational, historical, recreational, and scientific” benefits provided by maintaining a species presence throughout substantial proportions of its range (Carroll et al. 2010, Nelson et al. 2016). Science also increasingly supports the conclusion that preserving multiple populations furthers conservation efforts by enhancing adaptive potential, the genetic variability that allows species to adapt in the face of climate change and other factors altering Earth's ecosystems (Funk et al. 2019). The conservation of multiple genetically distinct ecotypes (i.e., populations adapted to a particular habitat) in a metapopulation structure across a species's range enhances metapopulation connectivity and allows gene flow and the exchange of adaptive variants among populations, enhancing the adaptive potential of the metapopulation as a whole (Crandall et al. 2000, Hoffmann and Sgro 2011, Hamilton and Miller 2015, vonHoldt et al. 2018, Hendricks et al. 2019a).

Quantitative models have been developed to predict how gene flow among populations enhances adaptive potential and reduces extinction risk in species experiencing environmental shifts because of climate change or other factors (Funk et al. 2019, Razgour et al. 2019). In addition, the conservation of adaptive potential has long been recognized as forming part of “an ethical imperative to provide for the continuation of evolutionary processes” (Soulé 1985), with value extending beyond its immediate role in lowering extinction risk over the relatively short time horizons typically considered in population viability analyses (Wolf et al. 2015).

## Conservation of intraspecific variation via the ESA's distinct population segment clause

Although the ESA predates the modern genetics research described above, lawmakers indicated their support for conserving intraspecific variation via the act's DPS and SPR clauses. Initially, almost all ESA listings were of entire species and subspecies, although the act did include language allowing listings of “any other group… in common spatial arrangement that interbreed when mature.” In 1978, Congress clarified the law to allow listing of “distinct population segments” (DPS) of vertebrate species (16 U.S.C. §1532(3.16)), although lawmakers directed that DPS designation be used “sparingly.”

**Figure 2.**
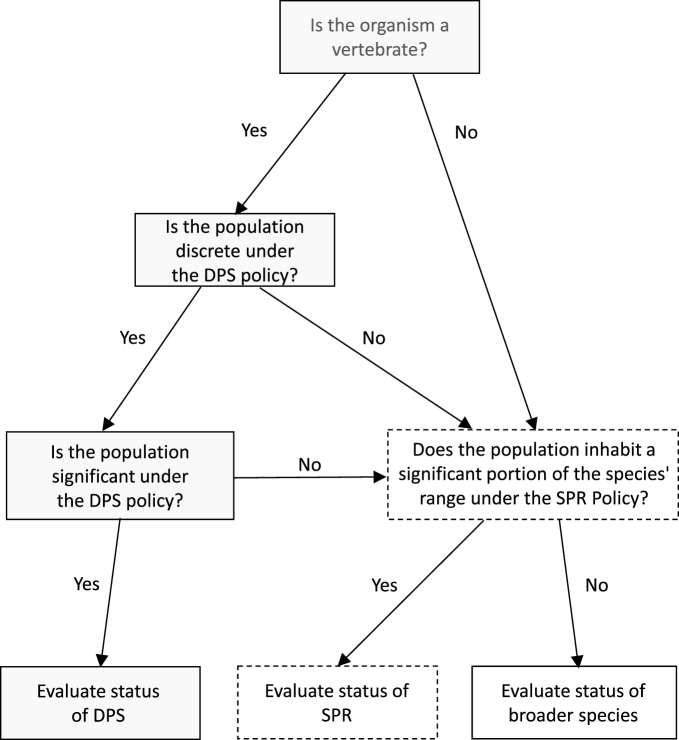
Flow diagram illustrating the proposed framework for designation of intraspecific conservation units under the US Endangered Species Act (ESA). Gray filled boxes represent decision steps currently taken when the Services evaluate whether a population constitutes a distinct population segment (DPS), a designation that the ESA limits to vertebrate species. Dashed boxes represented decision steps taken under the proposed “significant portion of range” (SPR) policy. Definitions of significance under the DPS and proposed SPR policy would be substantially similar but may diverge in emphasis as was described in the text.

In 1996, the Services (the US Fish and Wildlife Service [FWS] and its counterpart, the National Marine Fisheries Service [NMFS]) finalized a policy that evaluates a population's “discreteness” and “significance” to its taxon in order to decide whether the population qualifies for protection as a DPS (61 FR 4722; figure 2). Similar frameworks based on discreteness and significance were subsequently adopted outside of the United States, such as in Canada's policy for identifying designatable units within species and subspecies (COSEWIC 2018).

The DPS policy's factors for determining what constitutes a significant population include evidence that the population persists in a unique ecological setting, that the loss of the population would result in a significant gap in the range of the taxon, that the population represents the only surviving natural occurrence of a taxon that may exist as an introduced population outside its historical range, and that the population's genetic characteristics differ markedly from those of other populations (Waples et al. 2018). Discreteness requires either marked separation from other populations of the same taxon as a consequence of physical, physiological, ecological, or behavioral factors or delimitation by international governmental boundaries with important differences in management or conservation status (61 FR 4722). Recognizing that population connectivity rates fall along a continuum, the DPS policy's standard for a discrete population requires “marked” rather than complete separation (61 FR 4722). For example, NMFS identifies distinct populations for salmonid species even though a small proportion of returning fish will reproduce within adjacent regions rather than the natal population (Waples 2006).

## Recolonizing species and hybridizing lineages pose challenges for delineating intraspecific conservation units

A large proportion of litigation concerning conservation of intraspecific variation under the ESA (table 1) relates to gray wolf delisting. This is due not only to the fraught politics surrounding this species but also to aspects of its distribution and systematics. Defining intraspecific conservation units for species such as the wolf that have been extirpated from the majority of their historical range is more complex than for species that are declining but remain extant across their historical range. The range of a species is inherently difficult to define, being contingent on timeframe as well as spatial scale (Gaston and Fuller 2009). For example, when the FWS initially proposed to delist the gray wolf in 2013, no breeding pairs of wolves existed in California, and therefore, under the Services’ definition, the state was not within the species's range (78 FR 35664). However, by the time of the 2019 proposal, at least one breeding pair was known to inhabit California, and the FWS considered the state as within the species's range (84 FR 9653).

**Table 1.** A timeline of gray wolf listing and delisting related actions.

Year	Action	Conservation unit	Reference
1967	*C. l. lycaon* listed.	Subspecies	32 FR 4001, 11 March 1967
1973	*C. l. irremotus* listed.	Subspecies	38 FR 14678, 4 June 1973
1974	*C. l. lycaon* listed.	Subspecies	39 FR 1171, 4 January 1974
1976	*C. l. baileyi* listed as Endangered.	Subspecies	41 FR 17736, 28 April 1976
1976	*C. l. monstrabilis* listed as Endangered.	Subspecies	41 FR 24064, 14 June 1976
1978	*C. lupus* in lower 48 United States (except Minnesota) and Mexico reclassified as Endangered.	Species	43 FR 9607, 9 March 1978
1978	*C. lupus* in Minnesota reclassified as Threatened.	State population	43 FR 9607, 9 March 1978
2003	*C. lupus* Eastern, Western, and Southwestern DPS designated and reclassified.	DPS	68 FR 15804, 1 April 2003
2005	*C. lupus* DPS Rule vacated.	DPS	Defenders of Wildlife v. Norton, 354 F. Supp. 2d 1156 (D. Or. 2005); National Wildlife Federation v. Norton, 386 F. Supp. 2d 553 (D. Vt. 2005)
2007	*C. lupus* WGL DPS designated and delisted.	DPS	72 FR 6052, 8 February 2007
2008	*C. lupus* WGL delisting rule vacated.	DPS	Humane Society of the United States v. Kempthorne, 579 F. Supp. 2d 7 (D.D.C. 2008)
2008	*C. lupus* NRM DPS designated and delisted.	DPS	73 FR 10514, 27 February 2008
2008	*C. lupus* NRM Rule vacated.	DPS	Defenders of Wildlife v. Hall, 565 F. Supp. 2d 1160 (D. Mont. 2008)
2008	Protections for *C. lupus* WGL and NRM DPS reinstated.	DPS	73 FR 75356, 11 December 2008
2009	*C. lupus* WGL DPS designated and delisted.	DPS	74 FR 15070, 2 April 2009
2009	*C. lupus* WGL DPS delisting rule vacated.	DPS	Humane Society of the United States v. Salazar, 1:09–CV–1092–PLF (D.D.C. 2009)
2009	*C. lupus* NRM DPS (except Wyoming) designated and delisted.	DPS	74 FR 15123, 2 April 2009.
2009	Protections for *C. lupus* WGL DPS reinstated.	DPS	74 FR 47483, 16 September 2009
2010	*C. lupus* NRM DPS delisting rule vacated.	DPS	Defenders of Wildlife v. Salazar, 729 F. Supp. 2d 1207 (D. Mont. 2010)
2010	Protections for *C. lupus* NRM DPS reinstated.	DPS	75 FR 65574, 26 October 2010
2011	*C. lupus* NRM DPS delisted by Congress.	DPS	Public Law 112–10 and 76 FR 25590, May 5, 2011
2011	*C. lupus* WGL DPS designated and delisted.	DPS	76 FR 81666, 28 December 2011
2012	*C. lupus* in Wyoming delisted.	State population	77 FR 55530, 10 September 2012
2014	*C. lupus* WGL DPS delisting rule vacated.	DPS	Humane Society of the US v. Jewell, 76 F. Supp. 3d 69, 110 (D.D.C. 2014)
2014–2017	*C. lupu*s Wyoming delisting rule vacated but reinstated on appeal.	State population	Defenders of Wildlife v. Jewell, 68 F. Supp. 3d 193 (D.D.C. 2014), Defenders of Wildlife v. Zinke, 849 F.3d 1077 (D.C. Cir. 2017)
2013	Delisting of *C. lupus* in lower 48 United States (except NRM and WGL DPS) and Mexico proposed.	Species	78 FR 35664, 13 June 2013
2015	*C. l. baileyi* listed as endangered.	Subspecies	80 FR 2488 and 80 FR 2512, 16 January 2015
2015	Protections for *C. lupus* WGL DPS and C. lupus in Wyoming reinstated.	State population	80 FR 9218, 20 February 2015
2017	Delisting of *C. lupus* in Wyoming reinstated.	State population	82 FR 20284, 1 May 2017
2019	*C. lupu*s delisting in lower 48 United States (except NRM DPS and C. l. baileyi) and Mexico proposed.	Species	84 FR 9648, 15 March 2019

*Source:* Adapted from 2019 proposed delisting rule (84 FR 9648). *Abbreviations:* DPS, Distinct Population Segment; NRM, Northern Rocky Mountains; WGL, Western Great Lakes.

The conservation of such small recolonizing populations is important in part because their genetic composition can diverge rapidly from that of the source population, given the small number of founders. This divergence provides a rapid mechanism for novel and potentially adaptive genetic variants to originate and be acted on by natural selection. An example in North American wolves is provided by the historic spread of the allele controlling black coat color, which correlates with enhanced fitness during canine disease outbreaks (Schweizer et al. 2018).

In addition, canids such as the gray wolf can hybridize and form extensive zones of intergradation, which poses challenges for policies that involve assigning subspecies and genetic groupings to disjunct geographic areas (Leonard et al. 2005, vonHoldt et al. 2011). For example, the Great Lakes wolf population—on which the 2019 delisting rule depends for its claim that the gray wolf is recovered—is an admixture with contributions from up to three canid species (*C. lupus*, *Canis latrans*, and putative *Canis lycaon*; Heppenheimer et al. 2018).

Although the conservation of intergradation zones is important for maintaining adaptive potential (Leonard et al. 2005), populations in these areas may not meet the DPS policy's standard for discreteness (i.e., marked geographic or genetic separation). Recent genetic research has concluded that evolutionary relationships in canids and some other taxa resemble a web of life because of historical and possibly ongoing genetic exchange, rather than a tree of life defined by reproductive isolation (vonHoldt et al. 2018), implying that the discreteness standards in the DPS policy may not be well suited for protecting admixed populations important to the overall taxon. Such genomic admixture can be a rich source of beneficial alleles, which quickly boost genetic variation in recently bottlenecked populations (vonHoldt et al. 2018).

## Conservation of intraspecific variation via the ESA's significant portion of range clause

Lawmakers also included within the ESA a second clause supporting the conservation of intraspecific variation, which has proved more challenging for the Services to implement than was the DPS clause. The ESA of 1973 differed from two previous versions of the law (P.L. 89–669 [1966], P. L. 91–135 [1969]) in recognizing that endangerment has a geographic component and in extending legal protections to species “at risk of extinction throughout all or a significant portion of its range” (16 U.S.C. §1532(3.6)). The SPR clause suggests that Congress intended that managers interpret the concept of endangerment more broadly than an entire species facing the risk of extinction (Wolf et al. 2015). From this perspective, recovery requires not only that a species exist but also that it be present across all “significant” portions of its range (Carroll et al. 2010).

The ambiguity of the ESA's SPR clause, coupled with ongoing controversy concerning the geographic component of recovery under the statute, have led to numerous legal challenges to delisting proposals (table 1). Two related themes have emerged from the series of SPR-related court decisions, many of which involved the gray wolf. The first revolves around the meaning of the term *range* in the SPR clause. The courts, although deferring to the Services’ desire to interpret the term *range* as indicating current rather than historical range, have nonetheless required the agency to consider loss of historical range when assessing a species's viability (Enzler and Bruskotter 2009, *Humane Society v. Zinke,* 865 F. 3d 585 [2017]).

Second, in several decisions stretching over two decades (from *Defenders of Wildlife v. Norton,* 258 F. 3d 1136 [2001] to *Center for Biological Diversity v. Everson*, 1:15-cv-00477 [2020]), the courts have concluded that the Services must interpret the term *significant* in the SPR clause in such a way that it is not rendered duplicative; that is, a species in peril throughout all of its range must somehow differ from a species in danger of extinction throughout just a significant portion of its range (Enzler and Bruskotter 2009). Although the Services have made multiple attempts to establish policy defining SPR, several court decisions have concluded that the most recent (2014) SPR policy (79 FR 37577), like previous efforts, runs counter to congressional intent (*Humane Society of the United States v. Jewell,* Case No. 13–186 [2014]), and the policy has been vacated nationwide (*Desert Survivors v. US Dept. of the Interior,* 231 F. Supp. 3d 368 [2017]). The courts concluded that the 2014 policy did not distinguish between a species at risk in a SPR and one at risk throughout its range, because it made SPR status contingent on a conclusion that extirpation of a regional population would place the entire species at risk of endangerment in the relatively short timeframe represented by the Services’ definition of the “foreseeable future.”

## Recovery units as a tool for conserving intraspecific variation

The Services have also developed guidance for delineating “recovery units” as an additional tool for conserving intraspecific variation. A recovery unit is “a special unit of the listed entity that is geographically or otherwise identifiable and is essential to the recovery of the entire listed entity, i.e., recovery units are individually necessary to conserve genetic robustness, demographic robustness, important life history stages, or some other feature necessary for long-term sustainability of the entire listed entity” (NMFS 2018). The Services often evaluate whether a regional population merits recovery unit status on the basis of whether it contributes to a species's resiliency, redundancy, and representation (Evans et al. 2020 [preprint] doi:10.1101/2020.03.15.991174). These 3R criteria suggest that a species, to be considered recovered, should be present in many large populations arrayed across a range of ecological settings (Shaffer and Stein 2000). Recovery units are especially appropriate “for species occurring across wide ranges with multiple populations or varying ecological pressures in different parts of their range,” for “ensuring conservation of the breadth of a species's genetic variability… necessary to provide adaptive flexibility,” “reestablishing historical or maintaining current genetic flow,” and “encompassing current and historical population and habitat distributions” (NMFS 2018). The clause “necessary for long-term sustainability” is not strictly defined in the context of recovery unit designation. Nonetheless, unlike the invalid definition of SPR used in the Services’ 2014 policy, it is clearly distinct from the threshold used to judge whether a species is at risk of extinction throughout its range.

Although the existing recovery unit guidance provides a tool for conserving intraspecific variation, several shortcomings in its current implementation limit its effectiveness. The delineation of recovery units is discretionary, representing only about 2% of ESA-listed species, and is biased toward specific taxonomic groups (Evans et al. 2020 [preprint] doi:10.1101/2020.03.15.991174). Although the recovery unit guidance for defining intraspecific variants is relevant to defining SPR, the Services have not linked recovery units to the courts’ requirement that the agencies consider SPR in listing and delisting decisions. Although the recovery guidance states that “some recovery units may qualify as a DPS,” there is no clear decision tree to help planners decide which option to select (NMFS 2018). In theory, recovery units should inform consultations under the ESA's section 7 regarding whether an action by another federal agency places a species in jeopardy, but this frequently does not occur (Evans et al. 2020 [preprint] doi:10.1101/2020.03.15.991174).

The wolf example detailed below, in which the FWS proposed to delist a widely distributed species on the basis of the recovery of a single population (a proposal at odds with the practice for other species), reinforces the conclusion of Evans and colleagues ([preprint] doi:10.1101/2020.03.15.991174) as to the “need for standardized practice regarding the use of recovery units” (see box 1). We propose that explicitly linking the delineation of intraspecific conservation units to the ESA's SPR mandate would increase consistency, limit the broad discretion (and consequent opportunity for inappropriate political influence) that characterizes the Services’ current approach, and provide the foundation of an SPR policy that could withstand judicial review.

Box 1. **Distinguishing significant units within a species’ distribution.**

**Figure 3.**
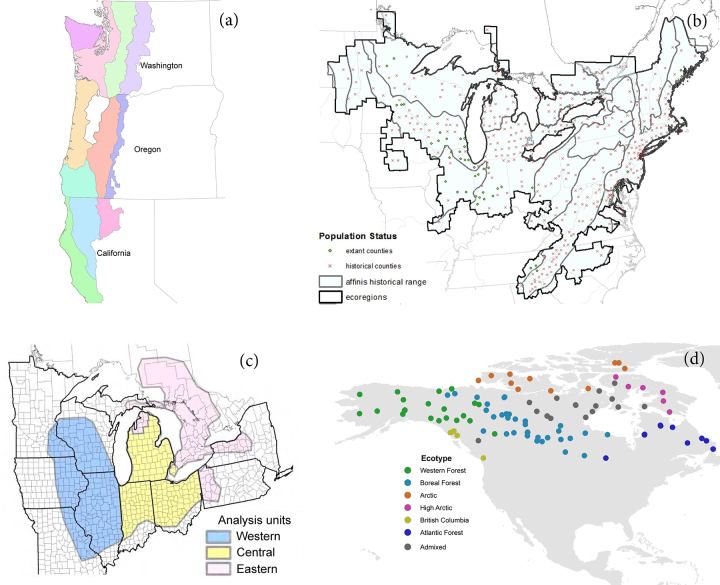
Examples of consideration of the significance of portions of a species's range in terms of their contributions to the species's adaptive potential: (a) the recovery plan for the Northern Spotted Owl (USFWS 2011) designated 11 recovery units on the basis of the physiographic provinces found within the species's distribution; (b) the status assessment for the rusty-patched bumblebee (Szymanski et al. 2016a) projected the number of representation units the species would inhabit under contrasting management scenarios; (c) the status assessment for the eastern massasauga rattlesnake (Szymanski et al. 2016b) identified three genetically distinct regional units needed to maintain the adaptive potential of the species; and (d) Schweizer and colleagues (2016) delineated six significant ecotypes for wolves inhabiting Canada and Alaska on the basis of associations between genetic clusters and 12 environmental variables. Source: (a–c) USFWS, (d) Rena Schweizer.

Federal agencies have employed a variety of approaches to identify intraspecific conservation units on the basis of how they contribute to a species's intraspecific variation and adaptive potential (Funk et al. 2019). Recovery units are often delineated on the basis of general ecosystem or habitat boundaries that are hypothesized to be relevant to adaptive variation in the species. The recovery plan for the northern spotted owl (*Strix occidentalis caurina*) designated 11 recovery units on the basis of the physiographic provinces found within the species's distribution (figure 3a; USFWS 2011). For species whose distribution has contracted, planners may consider the breadth of ecoregions encompassed by their historic distribution. The status assessment for the rusty-patched bumblebee (*Bombus affinis*) evaluated current and historical representation of the species in all ecoregions within its historical range, and projected the number of “representation units” (a surrogate for adaptive potential) that the species would inhabit under contrasting management scenarios (figure 3b; Szymanski et al. 2016a). Units can alternately be delineated on the basis of genetic data when such information is sufficient. In its status assessment of the eastern massasauga rattlesnake (*Sistrurus catenatus*), the FWS identified three genetically distinct regional units needed to maintain the adaptive potential of the species (figure 3c; Szymanski et al. 2016b).

Although many examples of delineating subspecific units are based solely on either genetic analysis or habitat discontinuities, stronger inferences can be drawn by using environmental data in combination with genetic information (Funk et al. 2012, Hendricks et al. 2019a). Landscape genomics techniques, such as selection tests and genotype-by-environment associations, provide powerful methods for distinguishing significant adaptive variants and ecotypes on the basis of the degree of adaptive differentiation between them (Carmichael et al. 2007, vonHoldt et al. 2011, Funk et al. 2012, Schweizer et al. 2016, Hendricks et al. 2019a).

Schweizer and colleagues (2016) were able to accurately infer the genetic cluster to which a gray wolf belonged on the basis of the habitat type (as defined by 12 environmental variables) where it was collected, allowing the delineation of six significant ecotypes for wolves inhabiting Canada and Alaska (figure 3d). Hendricks and colleagues (2019b) similarly used a maximum entropy approach to model distinct coastal and interior environmental niches for wolves in the US Pacific Northwest. The most relevant type of genetic information may differ when delineating DPS versus SPR. Intraspecific conservation units such as DPS, whose genetics have been shaped by both historical isolation (i.e., discreteness) and adaptive processes, can be delineated using both neutral loci and loci under selection (Funk et al. 2012). Other less-isolated but significant subunits of species (SPR) can be delineated primarily using loci that exhibit signatures of divergent selection (Funk et al. 2012).

The question remains as to how finely to divide a species's range—that is, how to discern “significant” intraspecific adaptive variants. DPS designation has been criticized as being partially subjective because there is no universally accepted threshold for the level of differentiation that confers evolutionary significance (Waples 1995). Although this criticism necessarily extends to identification of potential SPR, model selection metrics such as the Deviance Information Criterion (Gao et al. 2011) are frequently used to determine the best-supported number of clusters or subunits within a sample on the basis of genetic and environmental data. As Winker (2010) states, “the process of diagnosing states that exist along a continuum of differentiation can be difficult and contentious and necessarily has some arbitrariness; professional standards can be developed so that such diagnoses are objective.”

## Toward a consistent and effective framework for conserving intraspecific variation under the ESA

The current implementation of the ESA falls short in protecting intraspecific variation when faced with ecological and genetic complexities such as those described above. A more integrated approach to evaluating potential DPS and SPR can help overcome these challenges and prevent species such as the gray wolf from falling through the cracks. At first glance, the context of how *significant* is used in the SPR clause differs from how the term is used in the DPS policy. In the case of SPR, *significance* refers to a geographic area inhabited by a population (i.e., its range), whereas in the case of DPS, it refers to characteristics of the population itself. However, insights from landscape genetics, which maps population characteristics to environmental features, could allow the Services to interpret the term *significant* in a more consistent manner in relation to both the DPS policy and the SPR clause.

We propose a framework under which the relevant Service would consider both geography and genetics in assessing whether a population is in danger of extinction or likely to become endangered in the foreseeable future in a “significant” portion of its range. Figure 2 shows the decision tree that the framework envisions. If the Services were assessing a species that appeared to be under threat in only a portion of its range, but the species either was not a vertebrate or did not show marked isolation (i.e., discreteness), they would consider both of the following factors in assessing whether that portion of the species's range is significant: a) the geographic extent of the area in which the population is imperiled, compared with both the species's current and historical distributions and b) the current or potential future genetic distinctiveness and adaptive potential of the imperiled population.

This means of incorporating genetics into the assessment of SPR is also consistent with the DPS policy's consideration of a population segment's genetic characteristics compared with the species as a whole in assessing whether the population is “significant.” Such an approach in the context of assessing SPR would resemble current guidance on identifying recovery units (NMFS 2018) but would establish a consistent science-based policy linked to delisting rather than an ad hoc application of recovery guidance. By encompassing geography as well as genetics, this analytical approach allows the Services to also consider the range of “esthetic, ecological, educational, historical, recreational, and scientific” benefits cited in the ESA as provided by a species's presence across its range (Carroll et al. 2010). We consider in supplement S1 the related question of what regulatory actions follow if the Services find a species endangered or threatened within only a significant portion of its range.

Even if recolonizing populations of formerly widely distributed species have not yet diverged genetically, their significance can be evaluated in a forward-looking manner as contingent on a degree of differentiation great enough for evolutionarily important contrasts to accumulate in the future (Waples 2006). Bowen (1998) coined the term *geminate evolutionary unit* to describe a regional population that shows morphological, behavioral, or biogeographical differentiation but does not yet show genetic divergence at neutral loci. Such a population can be considered significant on the basis of its ability to contribute to future evolutionary potential—for example, because of colonization of a new habitat (e.g., as defined by ecoregions or climatic zones) with novel selective pressures. For example, the North Cascades region of Washington State, which may currently contain only transient grizzly bears, has nonetheless been the object of substantial recovery planning efforts in part because it represents a unique ecological and evolutionary context for the species within the contiguous United States (USFWS and NPS 2017).

The elements we propose the Services consider in identifying a “significant” portion of a species's range are also identified in the DPS policy as relevant to assessing a discrete population's significance. Therefore, it is feasible to coordinate the definitions of significance in the DPS and SPR policies. For example, recognition of evolutionary potential and the importance of geography are inherent in the DPS policy's consideration of the significance of unique ecological settings and potential gaps in a species's range created by the loss of a population, respectively. However, our framework (figure 2) allows for differences in emphasis and implementation to remain between the DPS and SPR policies’ definitions of significance. In addition, although existing guidance regarding recovery units informs our proposed SPR definition, the Services could retain the flexibility to identify recovery units for the purposes of recovery planning and implementation, even if such units were not identified as SPR.

## Gray wolf listing and delisting demonstrates the need for a consistent approach to conserving intraspecific variation

The several subspecies of North American gray wolf were among the earliest taxa listed as endangered under the ESA. The FWS shifted in 1978 to listing the wolf at the species level, except the Mexican wolf subspecies (*Canis lupus baileyi*), which remains listed separately (43 FR 9610). As wolf population numbers increased under ESA protection, the FWS repeatedly sought to remove some or all of the US population from the list of endangered and threatened species, only to be blocked by the courts in at least nine separate decisions since 2005 (table 1). The successive wolf delisting proposals have been characterized by scientific as well as legal controversy. The FWS withdrew a 2013 delisting proposal after a panel of scientific peer reviewers found flaws in the agency's taxonomic analysis (NCEAS 2014). A panel of invited scientific peer reviewers (including two of the present authors, CC and AT) also found significant shortcomings in the 2019 delisting proposal (Atkins 2019).

A notable feature of the successive delisting proposals is that they have varied widely in how they defined appropriate gray wolf conservation units, ranging from a focus on *C. lupus* as a whole to a focus on one or more DPS or populations inhabiting individual states (table 1). The most recent (2019) delisting proposal asserted that gray wolves in the contiguous United States (except for the separately listed *C. l. baileyi*) no longer merit ESA protection, on the basis of the premise that the agency can delist a species when a single regional population (in this case wolves inhabiting the Great Lakes states; figure 1) has recovered to a status the agency deems presently secure (84 FR 9683).

The roughly 4000 wolves estimated to inhabit the Great Lakes region constitute approximately two-thirds of the total population currently inhabiting the contiguous United States (figure 1). But is total population the only relevant metric for assessing the conservation status of a species? The Great Lakes population occupies only 3 of the at least 17 states within the species's historical range that hold substantial areas of habitat (figure 1). The approximately 2000 wolves inhabiting the Northern Rocky Mountain (NRM) region form the only other large regional population within the contiguous Untied States (figure 1). Because the US Congress passed legislation (Pub. L. No. 112-10, § 1713, 125 Stat. 38) removing ESA protections from the NRM population (the only instance of such legislative delisting since the ESA's passage), that population is counterintuitively not part of the listed entity considered in the 2019 proposal.

By arguing in the 2019 proposal that “wolves that occur outside the Great Lakes area… are not necessary for the recovered status of the gray wolf entity” (84 FR 9683), the FWS took a dramatic step away from its policy at the time it consolidated wolf subspecies into a single listing in 1978, when the agency offered “the firmest assurance that it will continue to recognize valid biological subspecies for purposes of its research and conservation programs” (43 FR 9610). The FWS's evolving position on wolf delisting exemplifies how the agency has moved away from Congress's vision of an ESA that protects intraspecific variation toward a more politically expedient approach predicated on a misrepresentation of the extent of intraspecific variation found in most geographically widespread species. For example, the FWS justified the central premise of the 2019 wolf delisting proposal—that wolf populations outside the Great Lakes region do not contribute to recovery—to a large degree on an assertion that the North American wolf population is genetically unstructured because the wolf's ability to disperse long distances would prevent genetic variation among subpopulations (84 FR 9685).

The development of high-throughput genotyping methods over the last decade has enabled an increasingly detailed analysis of historical and current population structure of North American wolves (Hendricks et al. 2019a). Wolf populations are now known to be characterized by complex genetic clines at several spatial scales, driven by historical biogeographic factors, isolation by distance, and association with particular ecosystems (Geffen et al. 2004, Carmichael et al. 2007, vonHoldt et al. 2011, Schweizer et al. 2016). Environmental factors related to climate zones significantly contribute toward genetic isolation by distance in North American gray wolves, likely through habitat matching decisions made by dispersers (Geffen et al. 2004). Environment factors, along with intraspecific competition for prime territories, resources, and access to reproduction, result in a nested structuring of genetic variation at both the continental and regional scales (Carmichael et al. 2007, vonHoldt et al. 2011, Schweizer et al. 2016).

## Distinct population segment policy as applied to the wolf

The highly structured North American wolf population revealed by genetic analyses has implications for determining whether conservation units below the species level are appropriate under the ESA (vonHoldt et al. 2011, Hendricks et al. 2019a). Wolf habitat in the contiguous United States is discontinuous enough to allow identification of DPS for some regional populations, despite occasional dispersal between regions (Carroll et al. 2006, CBD and HSUS 2018). For example, the FWS itself concluded in 2007 that despite “occasional individual wolves or packs [that] disperse among populations,” Northern Rocky Mountain wolves were markedly separated from other regional wolf populations (73 FR 10519).

In 2008, the FWS embarked on an effort to develop a comprehensive national strategy for gray wolf conservation by identifying appropriate wolf listing units within the broader continental distribution of the species (76 FR 26086). This national strategy was necessary because earlier proposals to remove protections for individual regional wolf populations by piecemeal designation and delisting of a single DPS within the larger range had been rejected by the courts (table 1; Alexander 2010). Five assessment units, including several potential DPS, were identified throughout the contiguous United States (figure 1). Although this closed-door process involving federal and state agencies lacked the inclusivity and scientific guidelines typical of recovery teams (PEER 2013), it nonetheless attempted (but never finalized) a comprehensive analysis of what recovery efforts might be appropriate in the different regions that include habitat for the species (Runge 2011).

In contrast, the 2019 proposed delisting rule did not attempt a comprehensive analysis of potential DPS status for regional populations but instead asserted that no regional wolf populations meet the DPS policy's standard for discreteness because the entire range of the gray wolf in the contiguous United States constitutes a single metapopulation (a term used in the rule in the broad sense of subpopulations linked by immigration and emigration). However, the Great Lakes and Pacific wolf populations, situated at the periphery of currently occupied wolf range, are separated by 1800 kilometers (km), much of which is transformed by agriculture. Although wolves inhabiting the Northern Rocky Mountains could provide an intermediate stepping stone population, any genetic interchange between these distant groups would necessarily be indirect and attenuated, allowing substantial genetic divergence (Schweizer et al. 2016).

The FWS has identified DPS for other large mammalian carnivores such as the grizzly bear (*Ursus arctos horribilis*) and does not claim that grizzly bear recovery in one region renders recovery efforts elsewhere unnecessary. Connectivity between regional grizzly bear populations, far from precluding DPS designation, has been judged by the FWS to be essential to long-term genetic health and recovery of those populations (82 FR 30502). The degree of genetic differentiation between regional wolf populations (e.g., between the NRM and Great Lakes populations) resembles that between grizzly bears inhabiting separate DPS in the Northern Rocky Mountains (vonHoldt et al. 2011, Cronin and MacNeil 2012).

Because average natal dispersal of male and female wolves (114 and 78 km; Boyd and Pletscher 1999) is several times that of male and female grizzly bears (42 and 14 km; Proctor et al. 2004), several grizzly bear DPS might occur within a single wolf DPS, as has been the case in the Northern Rocky Mountains. However, the entire gray wolf distribution in the contiguous United States cannot be considered a single genetically undifferentiated population, as was proposed in the 2019 delisting rule. The divergence in application of the DPS policy to grizzly bears and gray wolves demonstrates the need for a more consistent application of the DPS policy. Our proposed framework coordinating the DPS and SPR policies would not require modification of the existing DPS policy's criteria but, rather, their consistent application even to controversial species such as the wolf, enabled by strengthened support for scientific integrity from agency leadership (Carroll et al. 2017).

## Significant portion of range as applied to the wolf

While acknowledging the absence to date of a legally sufficient definition of SPR, the FWS in the 2019 proposed wolf rule attempted to satisfy future judicial review by evaluating whether regional populations outside the Great Lakes are “significant.” To support its claim that recovery of the Great Lakes population allows the agency to delist wolves throughout the contiguous United States, the FWS concluded that any currently listed wolf population found outside the Great Lakes region is not significant “because it is not biologically important” because of the small size of peripheral populations and the purported lack of genetic differentiation within the North American wolf population (84 FR 9648). This conclusion requires both a particularly narrow reading of the 3R criteria and a misrepresentation of research regarding wolf genetic population structure.

Under our proposed framework (figure 2), DPS could be identified for regional wolf populations that showed marked separation from other populations, whereas wolf populations inhabiting intergradation zones might instead qualify for delineation as SPR. The coastal Pacific Northwest (western Washington and Oregon and northern California; figure 1), one of the five regions assessed in the 2008 process, provides an example of a regional wolf population that meets the DPS discreteness criterion (figure 2). Marked separation can be established for this regional population as a consequence of several factors: physical (separation from larger inland populations by areas of nonhabitat), ecological (occupation of coastal rainforest ecosystems), genetic (unique genetic contributions from wolves from coastal British Columbia; Hendricks et al. 2019b), and an international governmental boundary separating US populations from coastal wolves in Canada that have different management status. Once discreteness has been established, wolves in the Pacific Northwest could merit significance because of their persistence in a unique ecological setting, which is used as a proxy for adaptive genetic differences, as well as the fact that loss of the population would result in a significant gap in the range of the taxon (Carroll et al. 2001, Waples et al. 2018).

The Colorado and Utah assessment unit considered in the 2008 process (figure 1), which historically formed a zone of intergradation between northern and southwestern wolf subspecies (Leonard et al. 2005), provides an example of an area that should be evaluated as a SPR, even if it is found to not show marked separation from adjacent populations (figure 2). This region, although currently supporting only a handful of wolves, represents a valid SPR because it holds abundant suitable habitat in a unique ecological setting (based on ecoregions or climate zones) subject to novel selective pressures (Carroll et al. 2006). Although we recognize that policy alone cannot ensure against inappropriate political influence in agency rulemaking, a coherent approach to DPS and SPR evaluation would be more likely to withstand litigation than the current ad hoc approach to wolf delisting, and more likely to result in the robust conservation outcomes envisioned by the lawmakers who drafted the ESA.

## Conclusions

In 2019, the US federal administration enacted sweeping changes to regulations interpreting the ESA that limit the statute's reach (83 FR 35174, Lambert 2019). The 2019 wolf delisting proposal forms part of this effort to advance a minimalist interpretation of the ESA's mandate, in that its central premise goes beyond what is necessary to support wolf delisting and seeks to establish a precedent that the ESA allows for a narrow view of what constitutes recovery of widely distributed species. By extending the assumptions of previous agency policy regarding the significant portion of range clause to their extreme, the proposed wolf delisting rule highlights the degree to which the conservation of intraspecific variation is central to ESA implementation and underlines the need to develop more effective policy concerning this issue. If applied generally to other species, the 2019 rule's approach to ESA implementation would represent a significant scaling back of recovery efforts for widely distributed species that would increase both short-term vulnerability and long-term loss of adaptive potential.

The recovery of formerly widely distributed species such as the wolf poses practical challenges for delisting and recovery planning (Treves and Bruskotter 2011). In some instances, an approach that requires continued federal management of the species throughout its range until the weakest regional population is secure may consume scarce conservation resources. An efficient strategy for recovery of such species could allow reduction of regulatory protections in regions that already hold abundant populations while maintaining protections in other regions that hold small recolonizing populations. The strategies we propose, based respectively on DPS and SPR designation, represent complementary approaches to achieving this flexibility that build on the Services’ existing standards for evaluating the significance of regional populations under the DPS policy and recovery unit guidance. Our proposed approach has relevance beyond the United States in the context of international regulations such as the European Union's Habitats Directive, which requires member states to achieve “favorable conservation status” for protected species without clarifying at what scale this status is to be achieved (Laikre et al. 2016).

When initially defining their resiliency, redundancy and representation criteria, Shaffer and Stein (2000) noted that successful conservation “will require identifying conservation targets not simply as species and communities but as the complexes of populations, communities, and environmental settings that are the true weave of biodiversity.” As advances in genomics increase our understanding of patterns of intraspecific variation, the conservation of adaptive potential merits increased emphasis as a key element in achieving the ESA's goal of “saving all the pieces” (Leopold 1968).

## Supplementary Material

biaa125_CarrolletalSITableS1TextS1

## Notes

Carlos Carroll (carlos@klamathconservation.org) is affiliated with the Klamath Center for Conservation Research, in Orleans, California. Daniel J. Rohlf is affiliated with the Earthrise Law Center, in the Lewis and Clark Law School, in Portland, Oregon. Bridgett M. vonHoldt is affiliated with the Department of Ecology and Evolutionary Biology, at Princeton University, in Princeton, New Jersey. Adrian Treves is affiliated with the Nelson Institute for Environmental Studies, at the University of Wisconsin, in Madison, Wisconsin. Sarah A. Hendricks is affiliated with the Institute for Bioinformatics and Evolutionary Studies, at the University of Idaho, in Moscow, Idaho.

## References

[bib1] Alexander K. 2010 Gray Wolves under the Endangered Species Act (ESA): Distinct Population Segments and Experimental Populations. Congressional Research Service. Report no. 49.

[bib2] America Atkins North 2019 Summary Report of Independent Peer Delisting Reviews for the US Fish and Wildlife Service Gray Wolf Delisting Review. Atkins North America. www.fws.gov/endangered/improving_esa/peer_review_process.html.

[bib3] Bowen BW. 1998 What is wrong with ESUs? The gap between evolutionary theory and conservation principles. Journal of Shellfish Research 17: 1355–1358.

[bib4] Boyd DK, Pletscher DH. 1999 Characteristics of dispersal in a colonizing wolf population in the central Rocky Mountains. Journal of Wildlife Management 63: 1094–1108.

[bib5] Carmichael LE, Krizan J, Nagy JA, Fuglei E, Dumond M, Johnson D, Veitch A, Berteaux D, Strobeck C 2007 Historical and ecological determinants of genetic structure in arctic canids. Molecular Ecology 16: 3466–3483. 17688546 10.1111/j.1365-294X.2007.03381.x

[bib6] Carroll C, Noss RF, Schumaker NH, Paquet PC 2001 Is the return of the wolf, wolverine, and grizzly bear to Oregon and California biologically feasible? Pages 25–46 in Maehr D, Noss RF, Larkin J, eds. Large Mammal Restoration: Ecological and Sociological Challenges in the 21st Century. Island Press.

[bib7] Carroll C, Phillips MK, Lopez-Gonzalez CA, Schumaker NH 2006 Defining recovery goals and strategies for endangered species: The wolf as a case study. BioScience 56: 25–37.

[bib8] Carroll C, Vucetich JA, Nelson MP, Rohlf DJ, Phillips MK 2010 Geography and recovery under the US Endangered Species Act. Conservation Biology 24: 395–403. 20151988 10.1111/j.1523-1739.2009.01435.x

[bib9] Carroll C et al. 2017 . Defending the scientific integrity of conservation‐policy processes. Conservation Biology 31: 967–975 28741747 10.1111/cobi.12958

[bib10] [CBD and HSUS] Center for Biological Diversity and Humane Society of the United States 2018 Petition to Maintain Protections for Gray Wolves (Canis lupus) in the Lower 48 States as Endangered or Threatened “Distinct Population Segments” under the Endangered Species Act. CBD www.biologicaldiversity.org/campaigns/gray_wolves/pdfs/Wolf-Petition-12-17-2018.pdf.

[bib11] Crandall K, Bininda-Emonds O, Mace G, Wayne RK 2000 Considering evolutionary processes in conservation biology. Trends in Ecology and Evolution 15: 290–295. 10856956 10.1016/s0169-5347(00)01876-0

[bib12] Cronin MA, MacNeil MD. 2012 Genetic relationships of extant brown bears (Ursus arctos) and polar bears (Ursus maritimus). Journal of Heredity 103: 873–881. 10.1093/jhered/ess090 23125409

[bib13] [COSEWIC] Committee on the Status of Endangered Wildlife in Canada 2018 Guidelines for Recognizing Designatable Units. COSEWIC cosewic.ca/index.php/en-ca/reports/preparing-status-reports/guidelines-recognizing-designatable-units.

[bib14] Enzler SA, Bruskotter JT. 2009 Contested definitions of endangered species: The controversy regarding how to interpret the phrase a significant portion of a species range. Virginia Environmental Law Journal 27: 1–67.

[bib15] Funk W, Forester BR, Converse SJ, Darst C, Morey S 2019 Improving conservation policy with genomics: A guide to integrating adaptive potential into US Endangered Species Act decisions for conservation practitioners and geneticists. Conservation Genetics 20: 115–134.

[bib16] Funk WC, McKay JK, Hohenlohe PA, Allendorf FW 2012 Harnessing genomics for delineating conservation units. Trends in Ecology and Evolution 27: 489–496. 22727017 10.1016/j.tree.2012.05.012 PMC4185076

[bib17] Gao H, Bryc K, Bustamante CD 2011 On identifying the optimal number of population clusters via the deviance information criterion. PLOS ONE 6: e21014. 21738600 10.1371/journal.pone.0021014 PMC3125185

[bib18] Gaston KJ, Fuller RA 2009 The sizes of species’ geographic ranges. Journal of Applied Ecology 46: 1–9.

[bib19] Geffen E, Anderson MJ, Wayne RK 2004 Climate and habitat barriers to dispersal in the highly mobile grey wolf. Molecular Ecology 13: 2481–2490. 15245420 10.1111/j.1365-294X.2004.02244.x

[bib20] Hamilton JA, Miller JM. 2015 Adaptive introgression as a resource for management and genetic conservation in a changing climate. Conservation Biology 30: 33–41. 26096581 10.1111/cobi.12574

[bib21] Hendricks SA, Schweizer RM, Wayne RK 2019a Conservation genomics illuminates the adaptive uniqueness of North American gray wolves. Conservation Genetics 20: 29–43.

[bib22] Hendricks SA et al. 2019b Natural re-colonization and admixture of wolves (*Canis lupus*) in the US Pacific Northwest: Challenges for the protection and management of rare and endangered taxa. Heredity 122: 133–149. 29880893 10.1038/s41437-018-0094-x PMC6327037

[bib23] Heppenheimer E et al. 2018 Population genomic analysis of North American eastern wolves (*Canis lycaon*) supports their conservation priority status. Genes 9: 606. 10.3390/genes9120606 PMC6316216 30518163

[bib24] Hoffmann AA, Sgrò CM. 2011 Climate change and evolutionary adaptation. Nature 470: 479–485. 21350480 10.1038/nature09670

[bib25] Laikre L, Olsson F, Jansson E, Hössjer O, Ryman N 2016 Metapopulation effective size and conservation genetic goals for the Fennoscandian wolf (*Canis lupus*) population. Heredity 117: 279. 27328654 10.1038/hdy.2016.44 PMC5026756

[bib26] Lambert J. 2019 Trump administration weakens Endangered Species Act. Nature (12 August 2019). www.nature.com/articles/d41586-019-02439-1. 10.1038/d41586-019-02439-1 32778705

[bib27] Leonard JA, Vila C, Wayne RK 2005 Legacy lost: Genetic variability and population size of extirpated US grey wolves (*Canis lupus*). Molecular Ecology 14: 9–17. 15643947 10.1111/j.1365-294X.2004.02389.x

[bib28] Leopold A. 1968 From A Sand County Almanac (1949). Oxford University Press.

[bib29] [NCEAS] National Center for Ecological Analysis and Synthesis 2014 Review of proposed rule regarding status of the wolf under the Endangered Species Act. NCEAS. www.fws.gov/science/pdf/Peer-Review-Report-of-Proposed-rule-regarding-wolves.pdf.

[bib30] Nelson MP, Vucetich JA, Bruskotter JT 2016 Ecological value and the US Endangered Species Act: Comment on Waples et al. (2015). Endangered Species Research 30: 187–190.

[bib31] [NMFS] National Marine Fisheries Service 2018 Interim Endangered and Threatened Species Recovery Planning Guidance. US Department of the Interior.

[bib32] [PEER] Public Employees for Environmental Responsibility 2013 Politics Dominated Wolf De-Listing Meetings: Science Was Afterthought in Developing Preferred Alternatives for Wolf Recovery. PEER. www.peer.org/news/news-releases/2013/06/26/politicsdominated-wolf-de-listing-meetings.

[bib33] Proctor MF, McLellan BN, Strobeck C, Barclay RMR 2004 Gender-specific dispersal distances of grizzly bears estimated by genetic analysis. Canadian Journal of Zoology 82: 1108–1118.

[bib34] Razgour O, Forester B, Taggart JB, Bekaert M, Juste J, Ibáñez C, Puechmaille SJ, Novella-Fernandez R, Alberdi A, Manel S 2019 Considering adaptive genetic variation in climate change vulnerability assessment reduces species range loss projections. Proceedings of the National Academy of Sciences 116: 10418–10423. 10.1073/pnas.1820663116 PMC6535011 31061126

[bib35] Runge M 2011 SW Wolf SDM Workshop: Background and Purpose. US Geological Survey Patuxent Wildlife Research Center www.peer.org/assets/docs/6_27_13_Wolf_Webinar.pdf.

[bib36] Schweizer RM, Vonholdt BM, Harrigan R, Knowles JC, Musiani M, Coltman D, Novembre J, Wayne RK 2016 Genetic subdivision and candidate genes under selection in North American grey wolves. Molecular Ecology 25: 380–402. 26333947 10.1111/mec.13364

[bib37] Schweizer RM et al. 2018 . Natural selection and origin of a melanistic allele in North American gray wolves. Molecular Biology and Evolution 35: 1190–1209. 29688543 10.1093/molbev/msy031 PMC6455901

[bib38] Shaffer ML, Stein BA. 2000 Safeguarding our precious heritage. Pages 301–321 in Shaffer ML, Stein BA, eds. Precious Heritage: The Status of Biodiversity in the United States. Oxford University Press.

[bib39] Soulé ME. 1985 What is conservation biology? BioScience 35: 727–734.

[bib40] Szymanski J, Smith T, Horton A, Parkin M, Ragan L, Masson G, Olson E, Gifford K, Hill L 2016a Rusty Patched Bumble Bee (*Bombus Affinis*) Species Status Assessment. US Fish and Wildlife Service.

[bib41] Szymanski J, Pollack C, Ragan L, Redmer M, Clemency L, Voorhies K, JaKa J 2016b Species Status Assessment for the Eastern Massasauga Rattlesnake (*Sistrurus Ca**t**enatus*). US Fish and Wildlife Service.

[bib42] Treves A, Bruskotter J. 2011 Gray wolf conservation at a crossroads. BioScience 61: 584–585.

[bib43] [USFWS and NPS] US Forest and Wildlife Service and National Parks Service 2017 North Cascades Ecosystem Draft Grizzly Bear Restoration Plan and Environmental Impact Statement. USFWS and NPS. parkplanning.nps.gov/projectHome.cfm?projectId=44144.

[bib44] [USFWS] US Fish and Wildlife Service 2011 Revised Recovery Plan for the Northern Spotted Owl (Strix occidentalis caurina). USFWS.

[bib45] [USFWS] US Fish and Wildlife Service 2019 Gray wolf Biological Report: Information on the Species in the Lower 48 United States. USFWS.

[bib46] vonHoldt BM, Brzeski KE, Wilcove DS, Rutledge LY 2018 Redefining the role of admixture and genomics in species conservation. Conservation Letters 11: e12371.

[bib47] vonHoldt BM et al. 2011 . A genome-wide perspective on the evolutionary history of enigmatic wolf-like canids. Genome Research 21: 1294–1305. 21566151 10.1101/gr.116301.110 PMC3149496

[bib48] Vucetich JA, Nelson MP. 2014 Conservation, or Curation? New York Times (20 August 2014) www.nytimes.com/2014/08/21/opinion/conservation-or-curation.html.

[bib49] Waples RS. 1995 Evolutionarily significant units and the conservation of biological diversity under the Endangered Species Act. American Fisheries Society Symposium 17: 8–27.

[bib50] Waples RS. 2006 Distinct population segments. Pages 127–149 in Scott JM, Goble DD, Davis FW, eds. The Endangered Species Act at Thirty: Conserving Biodiversity in Human-Dominated Landscapes. Island Press.

[bib51] Waples RS, Pacifici K, Kays R, Fredrickson RJ, Mills LS 2018 Is the red wolf a listable unit under the US Endangered Species Act? Journal of Heredity 109: 585–597. 10.1093/jhered/esy020 PMC6022562 29889268

[bib52] Winker K. 2010 Subspecies represent geographically partitioned variation, a gold mine of evolutionary biology, and a challenge for conservation. Ornithological Monographs 67: 6–23.

[bib53] Wolf S, Hartl B, Carroll C, Neel MC, Greenwald DN 2015 Beyond PVA: Why recovery under the Endangered Species Act is more than population viability. BioScience 65: 200–207.

