# Supplementary material for: Wolf Delisting Challenges Demonstrate Need for an Improved Framework for Conserving Intraspecific Variation under the Endangered Species Act
Source: Bioscience. 2020 Oct 29;71(1):73–84. doi: 10.1093/biosci/biaa125 (PMC7791361; doi:10.1093/biosci/biaa125)
Supplement: biaa125_CarrolletalSITableS1TextS1 [file biaa125_carrolletalsitables1texts1.pdf]

Supplementary Material for 'Wolf delisting challenges demonstrate need for improved framework for conserving intraspecific variation under the Endangered Species Act.'

Carlos Carroll[1], Daniel J. Rohlf[2], Bridgett M. vonHoldt[3], and Adrian Treves[4], and Sarah A. Hendricks[5].

1 Klamath Center for Conservation Research, Orleans, CA 95556 USA

2 Earthrise Law Center, Lewis and Clark Law School, Portland, OR 97219, U.S.A.

3 Department of Ecology & Evolutionary Biology, Princeton University, Princeton, NJ 08544, USA.

4 Nelson Institute for Environmental Studies, University of Wisconsin, Madison, WI 53706, USA.

5 Institute for Bioinformatics and Evolutionary Studies, University of Idaho, Moscow, ID, 83844, USA.

* carlos@klamathconservation.org

Supplementary Information Table S1. Definition of terms.

Distinct Population Segment (DPS) – A subspecific unit eligible for listing as a separate entity under the US Endangered Species Act. To qualify as a DPS, a population must be both discrete and significant. A population segment of a vertebrate species may be considered discrete if it satisfies either one of the following conditions: 1. It is markedly separated from other populations of the same taxon as a consequence of physical, physiological, ecological, or behavioral factors. Quantitative measures of genetic or morphological discontinuity may provide evidence of this separation. 2. It is delimited by international governmental boundaries within which differences in control of exploitation, management of habitat, conservation status, or regulatory mechanisms exist that are significant in light of section 4(a)(1)(D) of the Act. If a population segment is considered discrete under one or more of the above conditions, its biological and ecological significance will then be considered in light of the discrete population segment's importance to the taxon to which it belongs. This consideration may include, but is not limited to, the following: 1. Persistence of the discrete population segment in an ecological setting unusual or unique for the taxon, 2. Evidence that loss of the discrete population segment would result in a significant gap in the range of a taxon, 3. Evidence that the discrete population segment represents the only surviving natural occurrence of a taxon that may be more abundant elsewhere as an introduced population outside its historic range, or 4. Evidence that the discrete population segment differs markedly from other populations of the species in its genetic characteristics. Source: Policy Regarding the Recognition of Distinct Vertebrate Population Segments Under the Endangered Species Act. Federal Register 61:4722-4725.

Significant Portion of its Range (SPR) – An element of the definition of an endangered species included in the Endangered Species Act of 1973, stating that such species must be "at risk of extinction throughout all or a significant portion of its range". Source: Endangered Species Act of 1973. 16 U.S.C. §1532(3.6)). Under the framework proposed in this paper, the relevant Service (US Fish and Wildlife Service or National Marine Fisheries Service) would consider both

geography and genetics in assessing whether a population is in danger of extinction, or likely to become endangered in the foreseeable future, in a “significant” portion of its range. If the Service was assessing a species that appeared to be under threat in only a portion of its range, but the species either was not a vertebrate or did not meet the DPS policy’s standard for discreteness (Fig. 2), the Service would consider both of the following factors in assessing whether that portion of a species’ range is “significant”: a) the geographic extent of the area in which a population is imperiled, compared to both the species’ current and historical distribution; and b) the current or potential future genetic distinctiveness and adaptive potential of the imperiled population.

Recovery Unit (RU) – A special unit of the listed entity that is geographically or otherwise identifiable and is essential to the recovery of the entire listed entity, i.e., recovery units are individually necessary to conserve genetic robustness, demographic robustness, important life history stages, or some other feature necessary for long-term sustainability of the entire listed entity. The concept of a recovery unit is not mentioned in the Endangered Species Act, but has been developed by the Services to implement the mandate for recovery planning described in the Act. Source: National Marine Fisheries Service (2018). Interim endangered and threatened species recovery planning guidance.

Geminate Evolutionary Unit (GEU) - A population which shows morphological, behavioral, or biogeographical differentiation, but does not yet show genetic divergence at neutral loci. Such a population can be considered significant based on its ability to contribute to future evolutionary potential, e.g., due to colonization of a new habitat with novel selective pressures. Source: Bowen BW. 1998. What is wrong with ESUs? The gap between evolutionary theory and conservation principles. Journal of Shellfish Research 17:1355-1358.

Supplementary Information Text S1: Alternative approaches for regulatory actions consequent on finding a species endangered or threatened within a significant portion of its range

A challenge in interpreting the ESA's "significant portion of its range" (SPR) clause involves the question of what actions follow if the Service finds a species endangered or threatened within only a significant portion of its range. In 2007, the Department of Interior issued a legal opinion concluding that a species facing extinction within a SPR could be listed as threatened or endangered within that geographical area alone, meaning that the more secure populations of the species occurring outside the area would not receive ESA protection (79 FR 37577). However, this "SPR protects SPR" approach, which was later abandoned by the Service, could lead to problems if such listings decrease coordination of management strategies between neighboring jurisdictions and because protection of the larger metapopulation may be necessary to sustain threatened subpopulations (Gilpin 2012). Objections have also been raised that this approach because it extends DPS-like protections to plant and invertebrate taxa which the 1978 ESA amendments specifically excluded from DPS designation (SPR Team 2010).

The fact that the ESA only authorizes listing of species, subspecies, and vertebrate DPS may also imply that a species endangered within a SPR must be listed throughout its range (the "SPR protects species" approach). Under this interpretation, a SPR designation could result in more geographically-extensive protections than would listing of the same population as a DPS. Although counterintuitive, this may be appropriate if the SPR is dependent on demographic rescue from a larger region. Moreover, the ESA does include provisions (e.g., section 4(d) rules setting forth prohibitions applicable to threatened taxa, including restrictions on "take") allowing the Service to maintain a high level of protection within a SPR while limiting regulatory oversight outside of that region.

However, other sections of the ESA are more problematic under this approach. The ESA's Section 7 requires that federal agencies consult with the Service when their proposed actions potentially jeopardize protected taxa or adversely modify designated critical habitat. In 2019, the Services added into ESA regulations their interpretation that a management action can trigger a finding of adverse modification of critical habitat only in cases where the action

appreciably diminishes critical habitat *as a whole* (83 FR 35179). Under this policy, the critical habitat of a regional population within the SPR could conceivably be appreciably diminished without triggering an adverse Section 7 decision. Implementation of the "SPR protects species" approach would therefore require that Section 7 regulations be reformed to clarify that adverse modification should be evaluated based on threats to the SPR itself. Alternatively, the Services could use their authority to identify a population within a SPR as a recovery unit crucial to recovery of the species as a whole, allowing a finding of adverse modification based on adverse consequences to that recovery unit alone. In summary, both the "SPR protects SPR" and "SPR protects species" interpretations have practical implications under current policy which will require resolution via regulatory or policy changes absent amendment of the act itself (SPR Team 2010).

References

Gilpin M. 2012. Metapopulation dynamics: empirical and theoretical investigations. Academic Press.

SPR Team. 2010. White Paper: Options for Interpreting the phrase "Significant Portion of its Range". (30 August 2020; www.peer.org/assets/docs/noaa/6_21_2010_NMFS_White_Paper_on_SPR.pdf).

USDI [U.S. Department of the Interior]. 2007. The meaning of "in danger of extinction throughout all or a significant portion of its range." Memorandum M-37013. Office of the Solicitor, USDI, Washington, D.C.
